# Dental Education

**Published:** 1892-09

**Authors:** W. Xavier Sudduth


### DENTAL EDUCATION.[1]

BY W. XAVIER SUDDUTH, A.M., M.D., D.D.S.[2]

The question of the relation of dental colleges to the State laws governing the practice of dentistry in the United States has received considerable attention during the past year. It seems to me that nearly all who have taken part in the discussion have viewed the subject in the wrong light. Instead of its being the State laws *versus* the colleges, it is the colleges, or a part of them at least, *versus* the laws.

---

[1] Read before the American Academy of Dental Science, April 6, 1892.

[2] Dean of the College of Dentistry of the University of Minnesota.

42

The impression has gotten abroad that all the colleges belonging to the National Association of Dental Faculties are united in the opposition now being hurled at the State boards of dental examiners. This certainly is not the case in Minnesota. The law has no warmer supporters in this State than the Faculty of the College of Dentistry of the University of Minnesota. How many other colleges in the country look upon the question in this light I am unable to say, but I think that when the attitude of the different schools is canvassed it will be found that not a few are in favor of wise legislation on the subject. I do not think that the members of the boards should be brought into the discussion at all, except where it can be shown that they are not doing their duty. They are simply officers of the law, and have no option but to enforce it to the best of their ability, and they should have the hearty support of the people who enacted the law. As a rule, they are conscientious, capable men, who perform a thankless task at a considerable sacrifice both of time and means. From personal knowledge I know that such is the case with the members of the board in Minnesota. Feeling the injustice of much of the criticism passed upon them, we took occasion in our last announcement to state the position of our school in no uncertain language, as follows:

" The Faculty of this college recognizes the right of each State to control the practice of dentistry within its borders by regularly appointed boards of dental examiners."

There would be just as much injustice in abusing a policeman who should arrest you for breaking a well-known city ordinance, such as fast driving, for instance, as to blame the members of the boards for executing the law as they understand it. It is the law, then, that we have to deal with, and not the State boards.

What is the history of the enactment of the laws found on the statute-books of nearly all the States at the present time? It has been one of self-defence in nearly every case, for as time went by States without laws found themselves "becoming the dumping-ground for the charlatan and the ignoramus who had been driven out of other States." The enactment of laws governing the practice of dentistry in a few States forced all the rest to legislate against empiricism as a matter of self-defence.

As the standard of dental education has advanced, the laws enacted have, in most cases, become more rigid in their requirements, and many that passed laws early in the history of dental legislation are now finding them inoperative, to a greater or less degree, and are seeking to amend or entirely replace them. These laws are passed for the protection of the people, and not for the interest of dentists or dental colleges. A few of the States, recognizing the value of a college education, have thrown such restrictions around the privilege of examination as to debar all but college graduates from availing themselves of it; the medical law in Minnesota states explicitly that none but college graduates are admissible for examination, and prescribes the length of course which must have been pursued; while the dental law allows a little more leeway, yet it does virtually the same thing by making the time of credit for practice an increasing one from the date of the enactment of the law, which will in time debar all but college men from entering practice in the State.

New Jersey has also placed a premium upon a college education, and a large proportion of the States, in accepting college diplomas for registration without examination, have acknowledged the value of college instruction.

All these advances have been made by the several States voluntarily, under the impression that the colleges would appreciate them and fulfil the trust bestowed in them. The administration of the law in States that require all candidates to pass an examination has shown that not all incompetents were non-graduates, fully as large a proportion coming from the so-called reputable colleges.

It was found that a very large proportion of men presenting with diplomas were not, in the judgment of the members of the boards, safe men to intrust with the care of the mouths of the citizens of their State. The result has been wide-spread distrust in the honesty of the intentions of some of the colleges, and a growing impression that the only safe way to guard against the admission of incompetents is to require all candidates to pass an examination before being granted a license to commence practice.

What has brought about this change in the attitude of the State laws towards the colleges? Nothing else than the flagrant abuse of the confidence which was at first shown in the colleges. They are now only reaping the reward of a betrayed confidence. The seed of distrust has, however, been sown, and will, under the same law of self-defence which caused the enactment of the laws in the first instance, require the amendment of existing laws until all the States will require all candidates to pass a test examination. Thus are the many made to bear the sins of the few.

Should such a condition come to pass, it would be nothing more, however, than is required of our sister professions; laws for the control of the practice of law even more rigid than have been established in any State for the practice of medicine or dentistry have long been on our statute-books. Graduates of all our law schools must, after graduating, pass an examination. They must, in addition to legal fitness, possess and maintain a good moral character, or they can be "disbarred" at any time. If they desire to remove, they must obtain a certificate from the court before which they were admitted to practice for registration in another State. If they desire to practise in the Supreme Court of the United States they must sustain a good reputation for a specified time, and then pass a second examination to show their fitness for the higher courts. We hear nothing from Columbia, Harvard, or the University of Pennsylvania about the non-recognition of their legal diploma, and why should such an outcry be made against those States that see fit to require college graduates to pass an examination before granting them licenses to practise dentistry?

If a graduate of the College of Literature and Arts of Princeton, Yale, or the University of Pennsylvania desires to teach a district school in the backwoods in Illinois, he is required to pass an examination as to his fitness to do so before the county superintendent of schools, which must be repeated every two years. It is true that he may pass the State examination, which will give him immunity for five years, and still we hear nothing from their *alma mater* in behalf of these much-examined candidates who thus enter into competition for a share of the public funds. If, then, a legal or literary diploma does not carry any power to license the possessor to practise, teach, or preach, why should a dental diploma authorize its possessor to practise dentistry in any portion of the United States he may fancy, and become a perpetual license at that, as some would have it? What is there in dental education that makes a man non-amenable to the laws of the State in which he desires to practise? The situation is unique and plainly absurd on its face. There is no reason in asking that graduates in medicine or dentistry should be granted favors that are not accorded to law or literary graduates. Both the latter professions afford much greater opportunity for injury to the people than does the former.

If a man wants to employ a lawyer or a teacher, he can take his time to inquire into their qualifications, but if he gets sick or has a toothache, he has, many times, to employ the first one at hand and trust to luck for the results.

In that degree that a profession has to do with the public weal, so should the restrictions of law be thrown around its practice. A medical or dental diploma should represent the standard of education in the institution granting it, the same as a law or literary diploma, and no more. It should not carry with it the license to practise outside of the State in which the institution is located, and here in Minnesota we do not ask even that, but require graduates of the University to pass before the State Board just the same as other candidates, and, what is more, we find it beneficial in that it helps us to maintain a high standard in our own college.

To grant more than exemption from examination to its graduates in its own State would allow an institution to set the standard of education in other States, and would be in distinct violation of the right of each to control its own internal affairs. This privilege is one of the most cherished rights reserved to each State as it enters the Union. It has called forth more discussion than, perhaps, all the other clauses of the Constitution. More eloquence has been spent and more legal acumen brought to bear upon it than any question relating to State comity. There is no question that is guarded by the State more jealously than the right to regulate its own internal affairs, none that its citizens would sooner shed their blood for, except, perhaps, to defend the nation's interest. The close scrutiny and the legal discussions that have been handed down in the past have so defined its powers and limitations that it hardly seems possible that a break could be made in the claims made by the several States in this direction.

A quite recent decision in the United States Supreme Court bearing directly upon the point in hand was handed down in the "Original Package" discussion. In this case certain parties doing business under charters granted by the State of Illinois tried, under the head of "Inter-State Commerce," to force the sale of high wines and whiskeys in the original packages on the market in the State of Iowa when the people of that State had said by legal enactment that they did not want them. This was the last stronghold of the opposition, and unlimited means were expended by these wealthy corporations to gain their point. The decision was, however, adverse to their interests, the Court holding that the State of Iowa had not only the right, but the power, to control the liquor traffic within its own jurisdiction. Various other attempts have been made within the past few years under the same head, but invariably with the same result. The motive in each instance has been a mercenary one. Strong corporations have been willing to override the will and the good of the people in order that they might make a few more miserable dollars.

Let us see if we can find a parallel in the case in hand. Certain colleges doing business under charters granted them in the States in which they are situated find that the people of other States, for their own protection, are enacting laws that interfere with their business of producing dentists, in that said laws place a restriction on the absolute free entrance of their graduates into said States for the practice of their chosen profession, and they therefore threaten to force these States to give up their inalienable right of attending to their own internal matters, because in so doing they have run counter to the interests of a few private business corporations.

In what way have the laws interfered with their business? They have only said that the quality of the product of dental colleges, like that of other institutions, say creameries, must reach a certain grade or else it cannot find place on their markets.

Do the laws anywhere prevent the entrance of thoroughly qualified dentists into practice? Not in a single instance; on the other hand, such are heartily welcomed. If such is the case, then the inevitable result of the operation of dental laws will be to send a student to those institutions that can assure him such preparation as shall fit him to pass the dental board of the State of his choice, otherwise his diploma, obtained "at so much cost of time and labor," would be "worthless so far as permitting the holder thereof the right of practice." This would certainly be the case unless the individual could demonstrate that he was possessed of the qualifications which his diploma claims for him, and if he had them in his head and at his fingers' ends, it surely would be no great task for him to prove to any State board that he was really the individual named in the document. To do this would not require more than a few hours' time, and would surely be worth the effort just to get the signature of the secretary of the board, who is a legal representative of the law.

From some of the discussions on this question it would seem that there is doubt as to the "keeping qualities" of the product of some of our colleges. Granting that it is "A No. 1" when put out as shown by the document duly signed and sealed that is sent with each package, it might be well to establish a system of "rapid transit" from the "diploma mill" to the offices of the State boards in the several States; and as the product in some instances seems very likely to deteriorate in transit, as has been shown in the case of our own State examinations, where as high a proportion as five in nine failed to pass inspection, it does seem that it might be well to establish a system of "refrigerator cars," and for the moral influence it may have label each separate consignment "Original package."

But, jesting aside, the question as I look at it, when stripped of all prejudice, has a purely business aspect, to which let us turn our attention for a moment and see what light we can gain thereby. The strongest opposition to the enactment and enforcement of State laws governing the practice of dentistry is found in those schools that hold their charters as "private institutions," and who look upon their "plant" as a "vested right." They are in the business of turning out dentists, and they naturally desire to do so at the least expense possible, consequently are opposed on "business principles" to all State regulations that look towards the elevation of the standard. They went in with the rest for a three years' course because that played right into their pockets. Students must now remain one year longer in attendance, which means more time in the infirmary and an additional year's fees. But to raise the standard would require a selection of the material to be taught and better facilities for instruction, all of which would increase the expense and lessen the profit. The rejection of poor material by a higher entrance examination would lessen the number in attendance, and consequently cut down the *profits* of the infirmary. Then again, improved facilities would incur considerable expense, which would have to come *directly out of the profits* of said infirmary.

The plain statement of the facts of a case are never agreeable to all concerned, but it does seem to me to be about time to draw a line between the really professional schools and the "dental infirmaries." Exposures are never pleasant tasks, but when it is currently reported that one institution cleared sixteen thousand dollars in its infirmary during the session of 1889–90, and twenty thousand dollars in 1890–91, and that another reached the fabulous sum of thirty-six thousand dollars for 1888–89, and with the same management and an increased attendance of students in 1390–91 there was, in all probability, a proportionate increase in infirmary receipts, it does seem time that some one should call attention to the subject. Many other instances of the abuse of the dental infirmary might be cited, but these well-authenticated cases will suffice for my purpose.

I do not think there is any risk in saying that the cause for most of the opposition to State dental laws at the present time lies in the pernicious influence of our dental infirmary system. A mercenary spirit has crept into our colleges through the "open door" of the infirmary, in many instances completely overshadowing the primary object for which they were established.

A close analysis of the opposition to State dental laws will show any fair-minded man that the strength of the opposition bears a very close relation to the size of the balance-sheet of the infirmary of the college. This is quite natural, as the larger the profits the more at stake, and also the greater amount of ready cash on hand with which to obstruct dental legislation. In some instances this has not been confined to the bounds of their own State, as some institutions have gone outside and used means to prevent the passage of laws which the people of a sister State had framed in its interest, because the enactment of such laws would, to a greater or less degree, be inimical to the interests of said colleges.

The remedy for the situation lies in supporting just laws in every State in the United States. It is not essential that these laws should be uniform. Uniformity means a compromise, a lowering of the standard, and would militate against progress. Let each college do its duty by its students and come to the standard set by the most rigid law, and there will be no more need of complaint. It is impossible to harmonize the differences between the colleges and the State boards in any other way. The members of these boards have no option in the matter. It is their duty to execute the law as they interpret it. It has been said that at the meeting at Saratoga last summer the National Association of Dental Examiners "refused absolutely to be a party to any compromise." What else could they have done? They did not make the laws. What would "perfect unison" in such a case mean other than that the officers of the law had conspired to defeat the wishes of the people as expressed in the law? It has also been said "that a truce has been arranged for the present;" said truce is, however, only a "recommendation," and not mandatory upon the boards of the several States. The Board of Dental Examiners of the State of Minnesota has been compelled to send its resignation to the National Association because it could not comply with that portion of the recommendation referring to a partial report of the work of the board, our law being explicit on this point and requiring a full, detailed report.

I cannot see wherein "the interests of all are jeopardized by the present antagonistic position," as is held by some. I am sure the State boards can stand the antagonism. They have nothing to fear. So long as they faithfully perform their duty the people will stand by them, and if much more opposition is made to the execution of the law so as to increase the labors of the boards, they will vote them an increase in salaries. Outside opposition is the strongest argument to the people of the necessity of dental legislation. On the other hand, only good to the dental profession can come from a free discussion. The attitude of the different colleges will be shown, which will, in the end, work to the elevation of the standard of dental education. Some very plain words will pass before the question is settled. It is needless to look for any compromise steps to be taken by the States in the matter. The colleges might just as well accept the situation at once and take such action as will put them in harmony with the law. The interests of the many cannot be sacrificed for those of the few. I consider that the future is bright for the scientific advancement of dental education in this country.

